# Preclinical Evaluations of Modified Rice Hydrogel for Topical Ophthalmic Drug Delivery of Praziquantel on Avian Philophalmiasis

**DOI:** 10.3390/pharmaceutics13070952

**Published:** 2021-06-24

**Authors:** Treepecth Prompetch, Akawat Chailorm, Saruda Tiwananthagorn, Nithidol Buranapim, Siriporn Okonogi, Hirotomo Kato, Wasan Katip, Raktham Mektrirat

**Affiliations:** 1 Department of Veterinary Biosciences and Public Health, Faculty of Veterinary Medicine, Chiang Mai University, Chiang Mai 50100, Thailand; treepecth@gmail.com (T.P.); akawat.chailorm@gmail.com (A.C.); saruda.t@cmu.ac.th (S.T.); 2 Department of Companion Animal and Wildlife Clinic, Faculty of Veterinary Medicine, Chiang Mai University, Chiang Mai 50100, Thailand; nithidol.buranapim@cmu.ac.th; 3 Department of Pharmaceutical Sciences, Faculty of Pharmacy, Chiang Mai University, Chiang Mai 50200, Thailand; siriporn.okonogi@cmu.ac.th; 4 Research Center for Pharmaceutical Nanotechnology, Chiang Mai University, Chiang Mai 50200, Thailand; 5 Department of Infection and Immunity, Jichi Medical University, Tochigi 3290498, Japan; hirok@jichi.ac.jp; 6 Department of Pharmaceutical Care, Faculty of Pharmacy, Chiang Mai University, Chiang Mai 50200, Thailand

**Keywords:** praziquantel, rice, hydogel, ophthalmic, ostrich, *Philophthalmus gralli*

## Abstract

The present study aims to evaluate the efficacy of a novel drug delivery system of the modified rice hydrogel containing praziquantel (PZQ) against *Philophthalmus gralli* isolated from ostrich eyes and determine the toxicity of the preparation on chicken eye model. The parasiticidal activity of PZQ (0, 1, 10, and 100 µg/mL) was tested on *P. gralli*. The ophthalmic antiparasitic hydrogel was formulated with appropriate amount of PZQ and chemically modified rice gel. The parasitic morphology after exposure with the preparation was examined under scanning electron microscope (SEM). The anthelminthic efficacy of the preparation on motility and mortality of parasites was performed by visual inspection and vital dye staining. The ocular irritation of the preparation was evaluated for 21 days using standard avian model followed by OECD 405. The results demonstrated that the parasiticidal activity of PZQ against *P. gralli* appears to be in a concentration- and time-dependent manner. In addition, the concentration of PZQ 10 µg/mL (Chi squared test, *p* = 0.003) and exposure time for 24 h (log-rank test, *p* = 0.0004) is sufficient to kill parasites, when statistically compared to negative control group. Rice hydrogel containing a lethal concentration of 10 µg/mL PZQ was successfully prepared. The preparation illustrated good parasitic killing and motile inhibiting effect on *P. gralli* compared with PZQ 10 µg/mL and its control (*p* < 0.05). An appearance under SEM of non-viable parasite after being incubated with the preparation, showing parasitic deformity, was observed comparing with the viable parasite in 0.9% normal saline solution (NSS). Moreover, no irritation of chicken eyes was also observed. Our results contribute to understanding the efficacy and the safety of the rice hydrogel of PZQ which have a predictive value for controlling *P. gralli* on the animal eyes. However, the pharmacological application needs to be further investigated for the best possible therapeutic approach.

## 1. Introduction

*Philophthalmus gralli* (*P. gralli*), is digenean trematodes of the family Philophthalmidae occurring in the avian ocular infestations. In the past few years, a raising incidence of avian philophthalmosis have been reported in captive and wild birds. The epidemiological reports were commonly found in the tinamus major birds [1], the greater rhea [2], and the ostriches [3]. Moreover, the zoonotic philophthalmiasis in human cases has been also documented in various parts of the world, including Thailand [4]. The ostrich (*Struthio camelus*) is a valuable animal in the natural, the zoo, and the commercial husbandry sectors [5]. Additionally, this trematode was found in the ostriches, causing ocular swelling, conjunctivitis, constant lacrimation, and ocular purulent discharge [2,3]. Unfortunately, the therapeutic regimen with safety and efficacy profiles for the routine anthelminthic treatment of avian philophthalmiasis was not well recommended.

The several papers reporting successful chemotherapeutic clinical trials of the praziquantel (PZQ) in the treatment of human trematode and cestode infestations have been published. Interestingly, the effective therapeutic protocols of PZQ by single intramuscular medication for chicken philophthalmiasis were recently also reported [6]. However, the ocular delivery of parenteral drugs is limited by the blood–retinal barriers and ocular perfusion, since the animal eyes have a relatively low blood supply when compared to other parts of the body [7]. A previous study also reported that the topical treatment in patient ostriches with *P. gralli* infestation by the levamisole in combination with chloramphenicol ointment is more effectiveness than that from the parenteral treatments of the doramectin and the closantel [8]. Topical ophthalmic drug delivery has been contemplated to be a theoretical medication for ocular disorders directly related to the ocular anterior segment. Therefore, the non-invasive drug delivery development of PZQ has also been emphasized.

The gel preparation is one of the most preferable ophthalmic dosage forms because of its excellent adhesiveness, comfort, and easy removal throughout the eyes. Many efforts are being made to discover potential natural gelling agents. Thailand is a major producer and a world exporter of rice grain (*Oryza sativa* L.). Attempts have recently been made to turn this agricultural product into value-added pharmaceutical materials. The rice starch is an important biodegradable amylose and amylopectin [9,10]. Interestingly, the physicochemical characteristics of the modified rice powders can be feasibly made into good gelling agents in pharmaceutical gel preparation [11]. Moreover, the successful development of a mucoadhesive buccal film and an antiseptic hydrogel from rice for pharmaceutical delivery systems were previously published [12,13,14]. Hence, in developing anthelminthic preparation loading, the PZQ into modified rice gel should have more advantages for biological and pharmaceutical aspects.

The main aim of the present study is an attempt to evaluate pharmaceutical hydrogel preparation containing the biodegradable material carrier and the effective antiparasitic. In order to achieve this aim, the anthelminthic efficacy of the modified rice hydrogel of the PZQ against *P. gralli* was investigated. Moreover, the safety of the pharmaceutical preparation was also evaluated before further chemotherapeutic clinical trials.

## 2. Materials and Methods

### 2.1. Animals and Ethic Approval

The experiment was conducted in accordance with the protocols approved by the Animal Ethics Committee, Faculty of Veterinary Medicine, Chiang Mai University (Ethic Permit No. S12/2560, 28 August 2017). Three adult ostriches (15–20 years) with 80–120 kg body weight were included in the preclinical efficacy study. The captive birds were kept in a fenced pen with the environmental enrichment, the Chiang Mai Zoo, Thailand. The diet of birds was a mixture of the commercial ratite pellets, the roughages, and the supplements. Water was also provided ad libitum. For preclinical safety study, a total of ten Hy-Line Brown laying hens at 72 weeks of age were obtained from a commercial source. The chickens were placed in individual cages on the wire-mesh floor under controlled climate conditions in a poultry house of the Institute and exposed to a 14 L:10 D lighting schedule with a light intensity of 10 lux. During the experimental period, the water and the standard meal diet of laying hens were offered for ad libitum consumption.

### 2.2. Isolation of Ocular Flukes

Three ostriches enrolled in the present study performed at a similar age and body weight. The ostriches were administrated by intramuscular injection of with Xylazine 1–2 mg·kg^−1^ as pre-anesthetic agents. The avian patients were anesthetized Zoletil^®^ (Zolazepam and teletamine) 0.5–1 mg·kg^−1^ maintenance with 5% isoflurane [15,16,17]. Endotracheal intubation was carried out immediately after induction. Physiological parameters including the cloacal temperature, the heart rate and respiratory rate were observed. All ostriches were considered to be avian trematodiais on the basis of physical examination, with specific ophthalmic assessment of the ocular lesions. The critical parasitic infestation was semi-quantitatively evaluated, according to the following cross scale [18]. The parasitic samples were collected from both eyes of anesthetized ostriches. Care was taken throughout the sample collection to avoid any injury to the animals as the samples were taken non-invasively from the sterile cotton swab. The collected parasitic lavage was examined under an optical microscopy for the morphological identification of *P. gralli* [19].

### 2.3. Molecular Identification of P. gralli

The parasitic specimens for molecular study were fixed in 90% ethanol and submitted for DNA extraction. Genomic DNA (gDNA) was extracted using the NucleoSpin DNA RapidLyse Kit (Macherey-Nagel GmbH, Düren, Germany) according to the manufacturer’s instructions. The partial sequence of large 28S ribosomal RNA gene, approximately 1300 base pairs, was analyzed using forward primer digl2 (5′-AAGCATATCACTAAGCGG-3′) and reverse primer 1500R (5′-GCTATCCTGAGGGAAACTTCG-3′) with four internal primers including 300F (5′-CAAGTACCGTGAGGGAAAGTTG-3′) and 900F (5′-CCGTCTTGAAACACGGACCAAG-3′); internal reverse primers: 300R (5′-CAACTTTCCCTCACGGTACTTG-3′) and ECD2 (5′-CTTGGTCCGTGTTTCAAGACGGG-3′) PCR was performed following protocol described previously [20]. The cycle-sequenced using ABI BIGDYE™ (Foster City, CA, USA) chemistry, alcohol-precipitated, and run on an ABI PRISM 3100™ automated capillary sequencer. The obtained sequences were assembled and compared with the previously deposited in GenBank using the BLASTn program [21]. for specific diagnosis. Extraction and amplification control was done using *Fasciola gigantiga* [22], and distilled water was used as negative control.

### 2.4. Concentration–Response and Time-Kill Kinetics Assays of Praziquantel

Overall, 10 isolated *P. gralli* were transferred to individual wells of 24-well plates. For concentration–kill kinetics assay, the parasites were exposed to various concentrations of PZQ (0, 1, 10 and 100 µg/mL). The untreated control group was exposed to 0.9% normal saline, whereas the use of 0.25% Dimethyl sulfoxide (DMSO) as vehicle group was performed. The 24-well plate was incubated in the dark for 24 h. The parasiticidal activity of PZQ were observed under the optical microscope. For time-kill kinetics assay, the parasites were exposed to single concentration of 10 µg/mL PZQ for 0, 4, 8, 12, 16, 20, and 24 h. Death of a parasite was indicated by modified vital dye staining with 1% *w*/*v* methylene blue [23]. All assays consisted of at least three wells at each drug concentration and were also performed with three independent replicates. The number of dead parasites in each concentration was recorded.

### 2.5. Preparation of Modified Rice Hydrogel Base

All rice grains harvested during July–September 2016 were used. The glacial acetic acid, sodium hydroxide, monochloroacetic acid, and polysorbate 80 from Sigma Chemical Co. (St. Louis, MO, USA), dichloromethane and methanol from RCI Labscan Co., Ltd. (Bangkok, Thailand) were used for preparing the modified rice. A chemical modification method of the powder of rice grains was previously documented [12]. Briefly, the milled rice powder was subjected to etherification in a mixture of methanol–water medium. The proper amount of monochloroacetic acid was gently added. The dispersion was unceasingly stirred at 60 °C for 3 h and the solid phase obtained was then washed with 95% ethanol. The dried coarse sample was crushed into a fine powder by pulverizing machine and passed through an 80-mesh sieve. The modified rice powder was subjected to a hot air oven at 65 °C for 24 h to ensure the complete removal of alcohols, confirmed by GC analysis, before using for hydrogel base preparation.

### 2.6. Preparation of Rice Hydrogel Containing Praziquantel

An aqueous solution of the insoluble PZQ was prepared using the appropriate amount of polysorbate 80 as a solubilizing agent and modified rice gel as a viscosity building agent. After magnetic stirring, the mixture was subjected to three progressive heating and cooling cycles between 65 and 85 °C at 4 °C/min. Then, purified water was added to adjust the final concentration of polysorbate and PZQ to 20 µg/mL and 10 µg/mL, respectively. The mixture was gently stirred and then subjected to a high shear mixing device (Ultra-Turrax T25, IKA, Staufen im Breisgau, Germany) at high-speed stirring of 5000 rpm for 1 min. The rice hydrogel of PZQ was set overnight before confirming the desired viscosity of approximately 200 mPas using a Brookfield rheometer R/S-CPS (Middleboro, MA, USA). Then, the obtained formulation was adequately sterilized by autoclave maintaining at a temperature of 121 °C (200 kPa) for 15 min.

### 2.7. Anthelminthic Efficacy of the Hydrogel Formulation

Ten isolated *P. gralli* were transferred to individual wells of 24-well plates. The parasites were exposed to the rice hydrogel of PZQ 10 µg/mL. The untreated and the drug control groups were exposed to the 0.9% normal saline and PZQ 10 µg/mL, respectively. The 24-well plate was incubated in the dark for 12 and 24 h. Motility and mortality of parasites was performed by visual inspection and vital dye staining. The motility was evaluated according to 0–3 grades of ascending activity (0 = non motile with vital dye stain; 1 = no motile without vital dye stain; 2 = partial or slow motile; and 3 = normal or active motile). This experiment was triplicated, each performed with three independent replicates. The number of dead parasites was recorded, and the mortality rate was calculated.

### 2.8. Detection of Parasitic Morphology by Scanning Electron Microscope

The *P. gralli* in normal saline was incubated with the rice hydrogel of PZQ 10 µg/mL at 37 °C for 8 h. The specimen was preserved using 2.5% glutaraldehyde in phosphate buffer (pH 7.4) at 4 °C for 24 h. The specimen was dehydrated with graded ethanol and sputter coated with gold particles. The surface of the parasites was examined under SEM (JEOL JSM 5410LV, Tokyo, Japan). The viable parasites in 0.9% NSS without the formulation were used as a negative control.

### 2.9. Ocular Toxicity Study in Chickens

The ocular toxicity of the formulation was evaluated in chickens following internationally accepted guidelines (OECD Test Guideline 405) [24,25]. The chickens used in the experiment were randomly selected. A single high dose of 10 µg/mL PZQ in the modified rice hydrogel was administered by ophthalmic medication on the right side of chicken eye. The negative control group of the left side of chicken eye was treated in parallel with the 0.9% saline. All chickens were observed in detail periodically 1 h, 1, 2, 3, 7, 14, and 21 days for any ocular toxic effects, including cornea, iris, and conjunctiva [26]. Intake of water and food, along with body weight, were measured daily. Mortality, behavioral pattern, physical appearance changes, injuries, pain, and signs of illness were monitored daily during the period. After 21 days, all chickens were sacrificed, and their eyes were collected. The specimens were fixed in 10% neutral-buffered formalin and embedded in paraffin. The specimens were sectioned at 4 μm, deparaffinized with xylene, and hydrated with a graded alcohol series. The sections of the cornea were stained with Hematoxylin and Eosin (H&E, New York, NY, USA) and Periodic Acid-Schiff (PAS, Billerica, MA, USA) [27].

### 2.10. Statistical Analysis

Statistical analysis was performed using Stata software, version 14 (Stata-Corp, College Station, TX, USA). Descriptive statistics were used to report the results. The median and the interquartile range were used for continuous data, while the counts and the percentages were used for nominal data. All tests were two-sided and the significant values of each treatment compared with control group were calculated using the chi squared test. Differences between the untreated and treatment groups in rates of time to outcome, was compared using the Kaplan–Meier curve and the log-rank test for significance. The probabilities less than 0.05 were considered significant.

## 3. Results

### 3.1. Diagnosis of Avian Ocular Trematodiais and Identification of P. gralli

The avian patients 3/3 (100%) were diagnosed to be bilateral ocular trematodiais. The clinical signs including the ocular discharge and the blepharitis was observed. The parasites were visualized in the conjunctival sac and under third eyelid in 6/6 (100%) of eyes examined by direct ophthalmic examination. The parasitic burden of *P. gralli* was found in more than five parasites or presence of uncountable parasites in cluster. In addition, the eye of an animal classified as severe (+++) in the semi-quantitative analysis. The morphological characteristic of *P. gralli* was confirmed under an optical microscope. Moreover, the conjunctival trematode from the ostrich in the present study was identified as *P. gralli*, which was submitted to GenBank database under accession numbers MZ088139, 99.92% identity to *P. gralli* isolate from Greater rheas (Rhea Americana) in Arizona (JQ246434) [20].

### 3.2. Concentration–Response Parasiticidal Activity of Praziquantel

**Figure 1:**
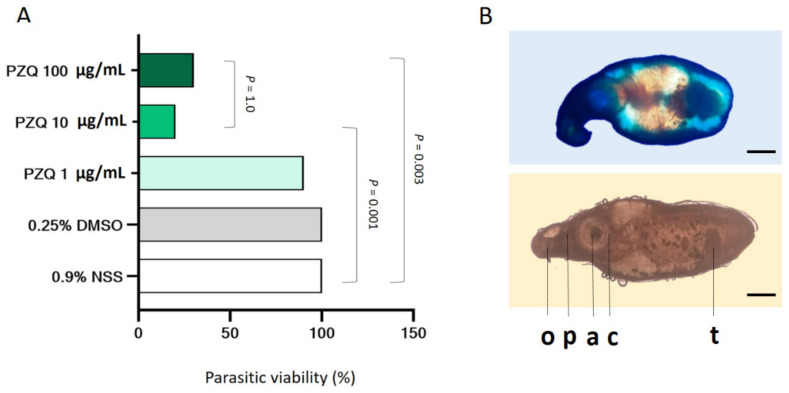
Dose-response parasiticidal activity of PZQ in *P. gralli.* The parasites were treated with a series of concentrations in the range 1–100 µg/mL for 24 h. (**A**) Data represent the percentages of parasitic viability (*n* = 10 per group). Experimental data were analyzed using the chi squared test. Significant *p*-values was highlighted onto the comparable bars (**B**) A non-viable parasite stained with vital dye of 1% *w*/*v* methylene blue (panels on the top side) and a viable parasite (panels on the bottom side) The scale bars represent 500 microns Features noted here include the oral sucker (**o**), pharynx (**p**), cirrus pouch (**c**), acetabulum (**a**), and testes (**t**).

The parasiticidal activity was determined at multiple concentrations of PZQ (1, 10, and 100 µg/mL) prior to evaluating its killing-kinetic effect. The parasite stained with vital dye of 1% *w*/*v* methylene blue were used as criteria to differentiate viable from non-viable samples (Figure 1). Our results revealed that the parasiticidal effect of PZQ were found to be dependent on drug strength. The mean viability of parasite at 24 h was obtained as shown in Figure 1. The results summarized that no death of parasite was observed in 0.9%NSS (negative control) and 0.25% DMSO (vehicle control) groups. A high viability rates (90%) of parasites exposed with 1 µg/mL of the of PZQ. Significantly decreasing viability rates (*p* < 0.05) were demonstrated in treatment at 10 and 100 µg/mL when compared with the negative control. However, the viability rates of PZQ 10µg/mL (20%) and 100 µg/mL (30%) was not a statistically significant difference.

### 3.3. Time-Kill Kinetic of Praziquantel

**Figure 2:**
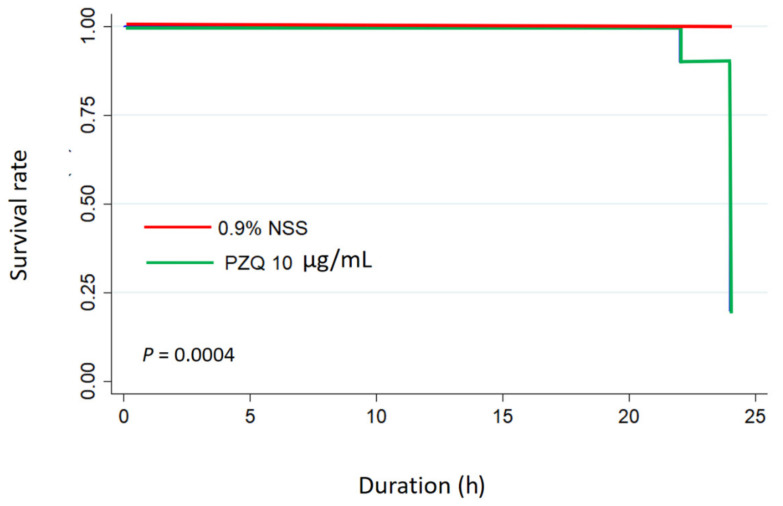
The parasiticidal activity of PZQ on time-killing in *P. gralli*. The parasites were exposed to PZQ 10 µg/mL and 0.9%NSS (control). Kaplan–Meier plot represents survival rate in two different groups (*n* = 10 per group). The Log Rank test was used for statistical analysis.

The study of concentration–response parasiticide suggested that a value corresponding to the optimum active concentration against in *P. gralli* are given at 10 µg/mL of PZQ. Therefore, the time-kill analysis of this concentration was only performed to evaluate the kinetic killing of in *P. gralli* at 0, 4, 8, 12, 16, 20, and 24 h. The Kaplan–Meier curve is used to demonstrate the survival time from a certain date to time of parasitic death (Figure 2). The result showed that the killing ability of PZQ 10 µg/mL was trendily performed in a time dependent manner. However, survival rate of *P. gralli* in the control groups were 100% throughout the trial period, whereas the parasitic survivability of PZQ 10 µg/mL group was markedly declined to 0.2 (20% survival rate) at 24-h post-exposure observation time (log-rank test, *p* = 0.0004).

### 3.4. Anthelminthic Efficacy of Hydrogel Containing Praziquantel

**Figure 3:**
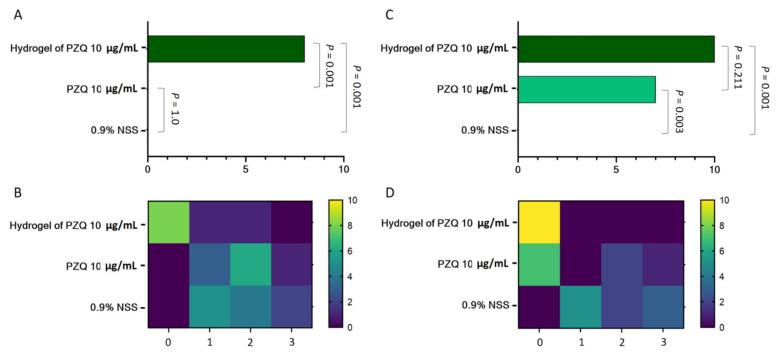
The anthelminthic efficacy of hydrogel of PZQ 10 µg/mL against *P. Gralli*. The parasites were treated with the formulation for 12 (panels on the left-hand side) and 24 h (panels on the right-hand side). The untreated and the drug control groups were exposed to the 0.9% normal saline and PZQ 10 µg/mL, respectively (*n* = 10 per group). Data represent the mortality rate of the parasites (**A**,**C**) and motility score (**B**,**D**). Experimental data were analyzed using the chi squared test. Significant *p*-values were highlighted onto the comparable bars.

The study of concentration–response and time-kill kinetics of PZQ suggested that a value corresponding to the concentration of PZQ 10 µg/mL is sufficient to kill parasites, when statistically compared to negative control group (*p* < 0.05). Rice hydrogel containing a lethal concentration of 10 µg/mL PZQ was successfully prepared. To examine the rice hydrogel containing 10 µg/mL PZQ induced antiparasitic efficacy, the mortality for *P. gralli.* was determined using vital dye staining and the motility score was also evaluated. The antiparasitic effect of the formulation on *P. gralli.* for 12 and 24 h was shown in Figure 3. Our results summarized that the antiparasitic effect of the formulation were found to be dependent on time. The mortality rate of the *P. gralli* from the control group was 0%, while that of the *P. gralli.* treated with the drug (10 µg/mL PZQ) and the formulation for 24 h was 70% and 10%, respectively. The formulation exhibited significantly higher mortality rate than that of the negative control in both time points (*p* < 0.05). On the other hand, the drug group was not significantly toxic to the parasite exposed for 12 h. By this assay, the motility score in a time-dependent manner was observed in all experimental groups. However, the results revealed that the formulation rapidly inhibited parasitic motility and also completely achieved zero grade of ascending activity at 24 h.

### 3.5. Scanning Electron Microscopic Analysis of Parasitic Morphology

**Figure 4:**
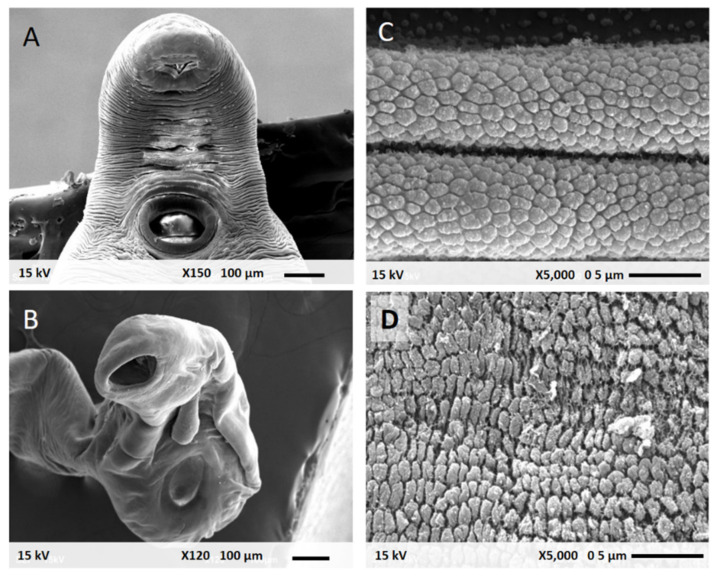
Scanning electron micrographs of *P. gralli* after incubation at 37 °C for 24 h; (**A**,**B**) Viable parasite in 0.9% NSS and (**C**,**D**) Non-viable parasite exposed to hydrogel of PZQ 10 µg/mL. Low 150× (panels on the left-hand side) and high magnification 5000× (panels on the right-hand side).

An appearance under SEM of viable *P. gralli* in 0.9%NSS was shown in Figure 4A. The untreated parasite demonstrated an elongated shape with normal acetabulum and oral sucker. A high magnification of typical morphology of *P. gralli* showed the smooth integument with flexibility in Figure 4C. The non-viable *P. gralli* incubated with hydrogel of PZQ 10 µg/mL was shown in Figure 4B. After incubation, *P. gralli* underwent significant morphological deformity with a withered shape. The parasitic surface clearly displayed flaccid and rough membrane structures.

### 3.6. Safety Property of Rice Hydrogel Containing Praziquantel

**Figure 5:**
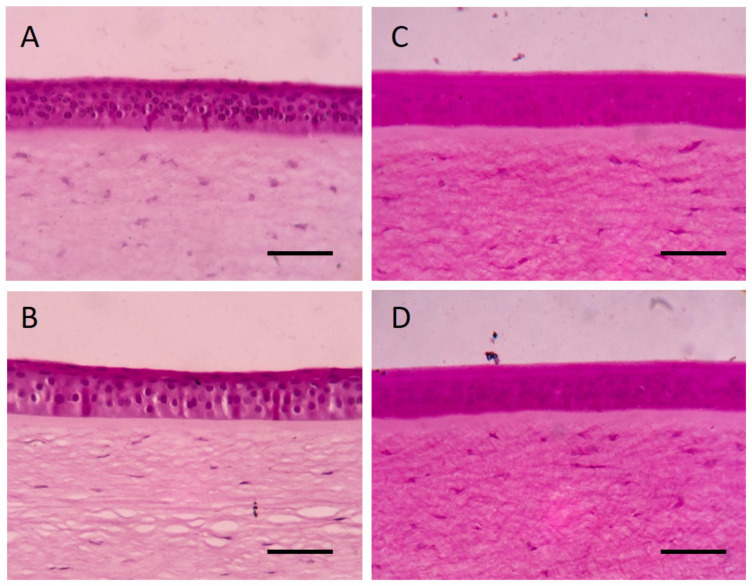
Histopathological findings of representative cross section from chicken corneas exposed to (**A**,**C**) negative control (0.9% NSS) and (**B**,**D**) Hydrogel of 10 µg/mL PZQ for 21 days. The images are representative of three independent assays (*n* = 3/chemical substance). Photomicrography with Hematoxylin and eosin (panels on the left-hand side) and Periodic Acid-Schiff staining (panels on the right-hand side) The scale bars represent 20 microns.

Ocular irritation assessment of chicken treated with the rice hydrogel containing 10 µg/mL PZQ revealed that no irritation of any animal occurred over 21 days. Some changes of general ocular appearance seemed to be found at 1–2 days after ophthalmic administration of the formulation, including the corneal opacity and the conjunctival redness. However, all the observed mild clinical changes were later recovered to normal (Table 1). Macroscopic evaluation of chicken eyes treated with the formulation found no characteristic changes that were different from the control group. The histopathological analysis revealed no significant structural differences between the treatment and control groups (Figure 5). The erosion, necrosis, and vacuolation of epithelium was not found. The normal stoma, bowman’s, and basement membrane integrity was also observed.

## 4. Discussion

All ostrich patients with the ocular discharge and the blepharitis were diagnosed as avian ocular trematodiais. These clinical findings were consistent with previous reports, in which the presence of several trematodes in the conjunctival sac and nictitating membrane of the infected ostriches caused ocular swelling, conjunctivitis, constant lacrimation and ocular purulent discharge, malnutrition, and physical weakness [2,3,5]. Furthermore, the phenotypic and the genotypic characteristics of *P. gralli* were confirmed by using the optical microscope and the direct sequencing of PCR product, respectively. The parasite sequences were found in GenBank, with accession numbers from Greater rheas (*Rhea Americana*) in Arizona [20]. These processes ensured the accuracy of *P. gralli* identity that was used in this study.

The dose-response and the time-killing kinetic of PZQ in *P. gralli.* were performed for prognosticating parasiticidal activity prior to develop the ophthalmic antiparasitic hydrogel with appropriate amount of PZQ and chemically modified rice gel. The results demonstrated that the parasiticidal activity of PZQ against *P. gralli* appears to be in a concentration- and time-dependent manner. In addition, the concentration of 10 µg/mL PZQ (Chi squared test, *p* = 0.003) and exposure time for 24 h (log-rank test, *p* = 0.0004) is sufficient to kill parasites, when statistically compared to negative control group. These findings are in agreement with previous studies in the parasiticidal activity of PZQ against trematode, in which the 40 μg/mL concentration of PZQ reduced viability of the *Schistosoma mekongi* by approximately the half maximal inhibitory concentration (IC50) [28]. The parasiticidal activity of prazquantel against cretodes, including Hymenolepis nana (1 µg/mL) for 5 min and Diphyllobothrium latum (0.1 µg/mL) for 4 h, was previously reported [29,30]. Interestingly, the effective therapeutic intramuscular medication of PZQ in patient chicken with *P. gralli* infestation were recently published [6]. Unfortunately, topical ophthalmic drug delivery of PZQ for avian ocular medication has not been studied scientifically.

The modify rice hydrogel containing a lethal concentration of 10 µg/mL PZQ was successfully prepared using the wet milling method. Since the native rice powder was not hydrated in water, the chemical modified starch by carboxymethylated etherification has been suggested [31]. The developed hydrogel formulation was not perfectly translucent. A prior study reported that gel turbidity resulted from the light scattering of particles entrapped inside the three-dimensional network of matrix in this dosage form [32]. In pharmaceutical formulations, mucoadhesive properties are essential for transmucosal application in both localized and systemic drug delivery systems [33]. The rheological property of obtained rice hydrogel was previously described as a pseudoplastic non-Newtonian flow [34]. Consequently, the rheological properties of both blank hydrogel base and the preparation of PZQ by thermal gelatinization occurs with the content and concentration of amylose [35]. The interfacial force of the rice hydrogel was affected from adsorption, wetting and diffusion phenomena [36,37]. The rice gel excipient was added due to their viscosity enhancing and adhesive properties, which can significantly improve the ocular retention time [38,39]. The mathematical modeling is a beneficial role to develop a novel pharmaceutical dosage form of rice hydrogel hydrogels for controlled drug delivery [40]. In addition, the behavior of PZQ release kinetics should be quantitatively evaluated with mathematical models using Higuchi model, and Korsmeyer-Peppas model [41,42]. Therefore, we anticipate an initial dose and the maintenance of effective concentration level of PZQ. However, the drug release mechanism of dextran hydrogel containing PZQ was previously reported. Accordingly, the alterations on swelling rate of dextran were able to influent in controlled release of the PZQ from polymeric matrix [43]. These findings are in agreement with the study on the solid dispersion of PZQ in sodium starch glycolate (SSG), which the release profile of PZQ was also associated with the capacity of SSG swelling [44]. Therefore, a likely expounding for the PZQ release system from formulation base of polysaccharides would be that the swelling capacity results in allowing for the entry of fluid into their particle diameter, increased by a chemical interaction between the polysaccharide chains.

The modified rice hydrogel of 10 µg/mL prazquantel illustrated good parasitic killing effect on *P. gralli* compared with the negative control group. Moreover, the preparation has proven more effective and has a more rapid onset of motile inhibiting action than the 10 µg/mL PZQ group. It is important to mention that the novel drug delivery system increases anthelminthic efficacy of PZQ in killing of *P. gralli*. Nevertheless, the various starch-based material incorporated antimicrobial agents can be a promising drug delivery system [45]. The enhanced antiparasitic efficacies of difference nanocarriers were recently also published [46,47]. Moreover, the photocatalytic degradation of PZQ was documented [48,49]. The photons of sunlight wavelengths are absorbed by starch polymer material [50,51]. Therefore, the degree of starch opacity may be suitable for applications in which there is light protection. As in a previous study, the thermoplastic corn starch (TPS) was able to absorb light of ultraviolet (UV) between 270 and 300 nm [52]. Moreover, the opacity of the rice starch (0.90 ± 0.07 A600/mm) was higher than that of the corn starch (00.61 ± 0.06 A600/mm) [53]. In addition, the photo-degradation of PZQ in rice hydrogel base might be more protected. Interestingly, an appearance under SEM of non-viable parasite after incubated with the preparation, showing parasitic deformity, was observed comparing with the viable parasite in 0.9% NSS. The previous experimental studies had suggested that a rapid influx of voltage-gated calcium channels accompanied by morphological changes of the parasite [54]. Current developments of ophthalmic rice hydrogel delivery of PZQ promise an improvement in overcoming the challenges posed by anterior segment diseases of avian philophthalmiasis.

As an initial step in drug development for ophthalmic medication, we performed an ocular irritation study in chickens to evaluate the safety of this pharmaceutical preparation in an in vivo model followed by OECD 405, which provides for toxic expression including general appearance and histopathological changes. Specifically, in our current study, each chicken received a single dose of ophthalmic rice hydrogel containing 10 µg/mL PZQ. No irritation in any chicken eyes was observed for 21 days. The temporary effects of the formulation on the avian eyes were found in some alterations including corneal opacity and conjunctival redness. However, all these signs disappeared to normal within 24–48 h post-administration. Microscopic evaluation by histopathology was used to verify the diagnosis of irritation or inflammation tissue. Hematoxylin and eosin staining was commonly used for routine pathological evaluation, whereas the periodic acid-Schiff reaction appeared to be the superior staining method for the chicken cornea [27,55]. Histopathology of the treated cornea revealed no significant structural differences between the treatment and control groups. These results are in line with studies of normal anatomical structure of the eyes, the corneal surface with smooth regular epithelium is uniform, and the structural framework of the cornea stoma are presented [56].

## 5. Conclusions

Our results highlighted the first efficacy and safety studies of the novel drug delivery system of the modified rice hydrogel containing antiparasitic PZQ. This present research has indicated that the parasiticidal activity of PZQ against *P. gralli* appears to be in the concentration- and time-dependent manners. Rice hydrogel containing a lethal concentration of 10 µg/mL PZQ was successfully prepared. The developed anthelminthic formulation exactly possesses a killing and motile inhibiting effect on *P. gralli*. The abnormal morphology of the treated parasite was also confirmed by SEM. This study also provides safety information of the formulation in the chicken eye model. However, further clinical studies are warranted to determine the best possible therapeutic approach.

## Figures and Tables

**Table 1:** The grades of ocular reactions (cornea, conjunctivae, and iris) from chicken eyes exposed to negative control (0.9%NSS) and Hydrogel of PZQ 10 µg/mL for 21 days.

Tested Groups	Animals	Corneal Opacity			Iritis			Conjunctival Redness			Conjunctival Redness			Total Score	Classification
		1	2	3	1	2	3	1	2	3	1	2	3		
0.9%NSS		0	0	0	0	0	0	0	0	0	0	0	0	0	Non-irritation
Hydrogel of PZQ 10 µg/mL		0.5	0	0	0	0	0	0.5	0	0	0	0	0	0.08	Non-irritation
